# Nanodisc single-molecule pulldown to study lipid-protein interactions

**DOI:** 10.1016/j.jlr.2025.100846

**Published:** 2025-06-20

**Authors:** Adriana Reyes-Ordoñez, Shweta Shree, Nilmani Singh, Stephen G. Sligar, Jie Chen

**Affiliations:** 1 Department of Cell and Developmental Biology, University of Illinois at Urbana-Champaign, Urbana, IL, USA; 2 Department of Biochemistry, University of Illinois at Urbana-Champaign, Urbana, IL, USA; 3 Carl R. Woese Institute for Genomic Biology, University of Illinois at Urbana-Champaign, Urbana, IL, USA; 4 Department of Chemistry, University of Illinois at Urbana-Champaign, Urbana, IL, USA; 5 Center for Biophysics and Computational Biology, University of Illinois at Urbana-Champaign, Urbana, IL, USA; 6 Cancer Center at Illinois, University of Illinois at Urbana-Champaign, Urbana, IL, USA; 7 Department of Biomedical and Translational Sciences, Carle Illinois College of Medicine, University of Illinois at Urbana-Champaign, Urbana, IL, USA

**Keywords:** AKT, lipid, Nanodisc, phosphatidylinositol phosphate, SiMPull, single-molecule

## Abstract

Beyond serving structural roles in the cell membrane, many phospholipids, including phosphatidylinositol phosphates (PIPs), are key signaling molecules that regulate a myriad of cellular processes. Specific interactions with PIPs are crucial for the functions of many signaling proteins, highlighting the need for a convenient and robust method to study lipid-protein interactions. Previously, we established a fluorescence microscopy-based lipid single-molecule pulldown (lipid-SiMPull) assay for detecting interactions between fluorescently tagged proteins of interest in whole-cell lysates and small unilamellar vesicles containing phospholipids of interest. Despite unique advantages of the lipid-SiMPull assay, small unilamellar vesicle is not an optimal membrane model due to its instability, heterogeneity in size, and a membrane curvature inconsistent with the relative flatness of the cell membrane. Here, we report the use of lipid Nanodiscs in lipid-SiMPull. Using PIP-protein pairs of known interactions, we show that Nanodiscs containing various PIPs can pull down protein targets specifically, with an estimated detection threshold of *K*_d_ in the 10–20 μM range. Remarkably, we find that each Nanodisc is bound by one copy of the protein (or protein dimer), conferring true single-molecule resolution to the assay. Transient interactions are characterized by the rebinding of proteins to individual Nanodiscs, and dissociation rates (*k*_off_) are determined from dwell time analysis. We apply this assay to interrogate structural requirements for the stability of AKT binding of PI(3,4,5)P_3_ and find that an intramolecular interaction between the PH domain and kinase domain is critical for stabilizing the AKT-PI(3,4,5)P_3_ interaction. This work estalishes the Nanodisc SiMPull assay as a simple and powerful approach for investigating protein-lipid interactions with single-molecule resolution.

In the cell, phospholipids serve not only as structural components of membranes but also as key signaling molecules. Interactions with phosphoinositides (PIs; phosphatidylinositol phosphate [PIP]) are crucial for the subcellular localization and activity of many proteins, impacting a wide range of signaling pathways (1, 2, 3). Despite extensive studies of PIPs in cell signaling, much remains to be explored in the molecular mechanisms of PIP-protein interactions. These interactions have been studied using a variety of biochemical and biophysical methods, such as surface plasmon resonance, liposome cosedimentation, isothermal titration calorimetry, and the most used albeit artifact-prone lipid overlay (lipid strips) assay (4, 5). The experimental methods can also be complemented by increasingly powerful computational approaches to reveal detailed mechanistic complexity of protein-lipid interactions (6).

A limitation of the experimental methods mentioned above is often the reliance on purified recombinant proteins, which are not always feasible or convenient to obtain. As a result, putative lipid-binding domains, rather than full-length proteins, are commonly used in lipid-binding studies. We previously established the lipid single-molecule pulldown (lipid-SiMPull) assay in which interactions between fluorescently tagged proteins of interest in mammalian cell lysates and specific PIPs in small unilamellar vesicles (SUVs) can be monitored via total internal reflection fluorescence (TIRF) microscopy (7). This assay circumvents the need for protein purification and potentially preserves the native states of proteins including post-translational modifications. Utilizing this assay, we have interrogated human PH domain-containing proteins and discovered an unexpectedly high degree of specificity of their binding to PIPs (8). Our study has also confirmed that isolated domains often do not recapitulate the lipid-binding properties of their full-length proteins.

Single-molecule studies have the unique power to decipher heterogeneous behaviors of individual biomolecules important for cellular processes, which can be masked by the average results in ensemble experiments (9, 10). Lipid-protein interactions have been studied with microscopy-based single-molecule approaches (11, 12). The current lipid-SiMPull assay using SUV offers single-vesicle but not single-molecule resolution. Even when a PIP is present at 5% of total lipids, there are ∼600 molecules of the PIP in a single 50-nm SUV, offering many opportunities for protein binding. TIRF microscopy cannot resolve multiple proteins bound to PIP in the same vesicle. For instance, we observed that each PI(3,4,5)P_3_-containing SUV recruited between 1 and 7 copies of the PH domain of AKT fused to GFP (7). This observation also highlights the heterogeneity of lipid vesicle size and/or PIP concentration in individual vesicles, a well-recognized complication in vesicle making. Another consideration is that SUV has a severe curvature that does not recapitulate the relative flatness of plasma membrane of the cell or the less pronounced curvatures of many intracellular membranes. Furthermore, membrane curvature in the cell is induced by the binding of various proteins (13) whereas the curvature of SUV is independent of any protein component. Therefore, SUV may not be the ideal membrane model to be used in lipid-protein binding assays.

Nanodiscs are self-assembled discoidal lipid bilayers that can provide a nanoscale native-like environment for studying protein-lipid interactions (14, 15). In addition to the relatively flat structure mimicking plasma membranes, Nanodiscs have several distinct advantages, including structural stability, precisely controlled size with well-defined lipid composition, and a small membrane area (under 10 nm if desired) that may accommodate only a single molecule of an average-sized protein, ideal for single-molecule studies. The current study establishes the use of Nanodiscs in lipid-SiMPull assay to achieve single-molecule resolution in studying lipid-protein interactions.

## Materials and Methods

### Plasmids

The following plasmids were obtained from Addgene: pEGFP-AKT-AH (plasmid #39533 (16)) and pEGFP-AKT (plasmid #39531 (16)) were gifts from Julian Downward; 2PH-PLCdelta-GFP was a gift from Sergio Grinstein (plasmid #35142); pJSK659 (EGFP-SnxA) was a gift from Jason King (plasmid #205128 (17)); and p40PHOX-PX-EGFP was a gift from Michael Yaffe (plasmid #19010 (18)). EGFP-AKT mutants (W80A, Q218A, T450A, and F469A) and EGFP-p40PHOX-PX mutants (R60A and K92A) were created using the QuikChange Lightning Site-Directed Mutagenesis kit (Agilent).

### Nanodisc preparation

Nanodisc self-assembly was performed using previously described protocols with slight modifications (15). Nanodiscs were assembled with varying molar ratios of 1,2-dimyristoyl-*sn*-glycero-3-phosphocholine (DMPC), 1,2-dimyristoyl-*sn*-glycero-3-phosphoserine (DMPS), and PIs (PI(3,4,5)P_3_, PI(4,5)P_2_, PI(3,5)P_2_, and PI3P). Briefly, lipids dissolved in chloroform were dried under vacuum overnight and resuspended in 200 mM sodium cholate. The membrane scaffold protein MSP1D1, expressed in *Escherichia coli* BL21 Gold (DE3) and purified as described earlier (19), was biotinylated at the C terminus. Biotinylated MSP1D1 was mixed with solubilized lipids at appropriate lipid-to-protein molar ratios depending on the lipid composition. The mixture was incubated for 15 min at room temperature. To initiate Nanodisc formation, Amberlite XAD hydrophobic beads were added to remove detergent, and the mixture was incubated overnight. Assembled Nanodiscs were purified using a Superdex 200 Increase size-exclusion chromatography column (GE Healthcare) equilibrated with 1× PBS. Nanodiscs containing 15% DMPS and 5% PIP (or 10% PIP_3_) in a DMPC background were prepared. Negative control Nanodiscs containing only 15% DMPS in a DMPC background were also prepared. The percentages of phospholipid composition indicate the number of anionic lipid molecules per total lipid molecules in the Nanodiscs, that is, 15% DMPS Nanodiscs have about 12 DMPS molecules per leaflet (total lipid molecules per leaflet ∼80 lipids). Prepared Nanodiscs were stored in aliquots at −80°C. To aid visualization by microscopy, Nanodiscs were incubated at 37°C for 1 h with 1,1′-dioctadecyl-3,3,3′,3′-tetramethylindodicarbocyanine (DiD) at 10:1 M ratio (DiD:disc) for dye incorporation (20). Under these conditions, we determined that most of the Nanodiscs contained two molecules of DiD per disc (e.g., see supplemental Fig. S1), which was ∼1% of DiD relative to total Nanodisc mass. Labeled discs were stored at 4°C until use and diluted with 10 mM Tris-HCl (pH 8.0) and 150 mM NaCl to achieve a density of ∼700 discs/microscopy field.

### Cell culture, transfection, and cell lysate preparation

Human embryonic kidney 293 cells were cultured in high-glucose DMEM supplemented with 10% fetal bovine serum, 2 mM l-glutamine, and penicillin-streptomycin at 37^o^C in 5% CO_2_. For transfection, cells were seeded in 6-well plates and transfected at 60–70% confluence with 2 μg of plasmid DNA and 6 μg of polyethylenimine. The DNA-polyethylenimine complex was incubated at room temperature for 15 min before being added to the cells. After 16–24 h, cells were washed once with ice-cold PBS and resuspended with 300 μl per well of detergent-free buffer (40 mM Hepes, pH 8.0, 150 mM NaCl, 10 mM ß-glycerophosphate, 10 mM sodium pyrophosphate, 2 mM EDTA, and 1x Sigma protease inhibitor cocktail). Cells were lysed by probe sonication for 3 s on ice followed by ultracentrifugation at 90,000 *g*. EGFP concentration in lysates was measured in a plate reader (Agilent, BioTek Synergy LX) at excitation 488/emission 520 and calculated using a standard curve of pure recombinant EGFP (ProSpec Bio; #PRO-1606). Each cell lysate was diluted in vesicle buffer (10 mM Tris-HCl, pH 8.0, 150 mM NaCl) to 5 nM EGFP for the assays.

### Lipid-SiMPull assay

The assay was modified from previously reported procedures (7). Quartz slides were thoroughly cleaned and passivated with PEG doped with 0.1–0.2% biotin-PEG. Prior to the assay, each slide was separated into six chambers using double-sided tape (Scotch 3M) and Epoxy (Loctite), and then each slide chamber was incubated with 200 μg/ml NeutrAvidin (Invitrogen; #A2666) for 10 min. Biotinylated Nanodiscs were loaded into the slide chambers and incubated for 10 min, followed by washing with vesicle buffer. Freshly prepared whole-cell lysates were flowed into slide chambers (80 μl lysate per chamber) and incubated for 20–30 min at room temperature. An inverted TIRF microscope equipped with Olympus 100x NA 1.4 lens and an EMCCD camera (Andor iXon Ultra 897) was used to acquire single-molecule data at 10 frames/second. Diode pumped solid state lasers were used to excite EGFP at 488 nm (Coherent) and DiD at 638 nm (Cobalt).

### Image processing and data analysis

TIRF 20-frame movie acquisitions were processed in IDL (Edition 6.2) to generate image files, and the number of fluorescent spots was identified with point-spread function. MATLAB, version 8.4.0.150421 (R2014b) was used to quantify the identified spots. The number of EGFP spots collected prior to addition of cell lysate (background) was subtracted from the number of EGFP spots after lysate addition to generate the data for each image. At least 10 microscopy images (1,600 μm^2^ each) were analyzed to yield the average number of spots per imaging area for each assay. MATLAB was also used to classify the fluorescence trace of each molecule for having one to four photobleaching steps and to measure dwell time of transient binding events. Pooled dwell time data from three independent experiments were analyzed using maximum likelihood estimation with a single, uncensored exponential decay model to yield dissociation rate constant (*k*_off_) (21, 22). Standard error (SEM) and 95% confidence intervals were also derived from this analysis.

### Statistical analysis

**Fig. 1:**
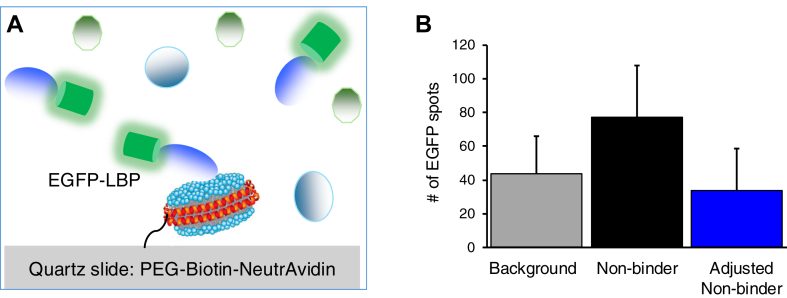
Lipid-SiMPull assay using Nanodiscs. A: Graphical representation of the assay: biotinylated Nanodiscs containing a lipid of interest are tethered via neutravidin to passivated quartz slides coated with biotinylated PEG; cell lysates containing an EGFP-tagged protein of interest are flowed into the slide chamber. After incubation, TIRF microscopy is performed to visualize Nanodiscs and associated EGFP proteins. B: Determining assay threshold: EGFP-fusion proteins were transiently expressed in HEK293 cells, and cell lysates (5 nM EGFP-fusion) were subjected to SiMPull assay with Nanodiscs containing various PIP or no PIP. EGPF spots on Nanodisc-tethered slides were quantified before (background) and after incubation with cell lysates containing EGFP fusion of a protein known to not bind the PIP in the Nanodiscs or negative control Nanodisc (nonbinder). The value of “adjusted nonbinder” was obtained by subtracting “background” from “nonbinder” in each assay. Data shown are mean ± SD for 175 assays of lipid-protein pairs known to not interact. Detection threshold is determined to be 80 (mean adjusted nonbinder + 2x SD).

One-sample one-tailed *t*-test was performed to compare SiMPull data (EGFP spots) to a predetermined threshold for binding (Fig. 1B). Unpaired two-tailed *t*-test was performed to compare data of percentage of rebinding. To compare *k*_off_ values, a likelihood ratio test (LRT) was performed, where the calculated LRT statistic was compared with a Chi-square distribution with 1 degree of freedom. For all statistical analyses, *P* < 0.05 was considered significant.

## Results

### Validation of lipid-SiMPull assay with Nanodiscs for known PIP-protein interactions

For the lipid-SiMPull assay, biotinylated Nanodiscs (∼10-nm diameter) containing various PIPs were immobilized onto slides, and human embryonic kidney 293 cell lysates containing 5 nM transiently expressed EGFP-fusion proteins were flowed into the slide chambers followed by TIRF microscopy to detect PIP-protein interaction (Fig. 1A). To validate the assay, we selected four protein domains that had been previously characterized for their specificity in PIP binding: AKT-PH (23), PLCδ-2xPH (24), p40PHOX-PX (18), and the recently reported SnxA (17). PIP Nanodiscs contained PI(3,4,5)P_3_, PI(4,5)P_2_, PI(3,5)P_2,_ or PI3P. As a negative control, Nanodiscs contained no PIP. Each of the four proteins was assayed against each of the five types of Nanodiscs. Pulldown of EGFP proteins was quantified by counting green fluorescence spots, and these data were collected from three independent experiments for each protein-lipid pair. To set a threshold for lipid binding, the number of EGFP spots per 1,600 μm^2^ imaging area after subtraction of no-lysate background was compiled for 175 assays of expected negative controls (e.g., any of the proteins with PIP-free Nanodisc, AKT-PH with PI(4,5)P_2_, etc.) as shown in Figure 1B. We arrived at 80 by taking mean + 2x standard deviation as the threshold for binding. As with any SiMPull assay, data would be interpreted as a binary outcome—binding or no binding.

**Fig. 2:**
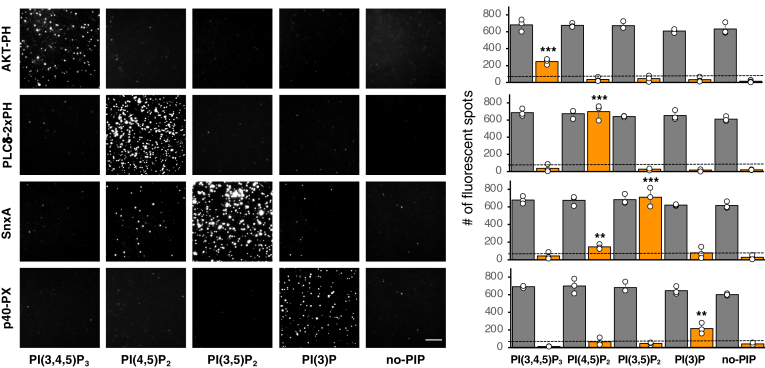
Validation of lipid-SiMPull assay with Nanodiscs for known PIP-protein interactions. EGFP-fusion proteins were transiently expressed in HEK293 cells, and cell lysates (5 nM EGFP-fusion) were subjected to SiMPull assay using Nanodiscs containing 5% PI(3)P, PI(4,5)P_2_, PI(3,5)P_2_, or PI(3,4,5)P_3_, or 0% PIP as negative control (no-PIP). Representative TIRF images are shown. Scale bar represents 10 μm. The graphs show data of average number of adjusted EGFP spots per image area. Gray bars: DiD; orange bars: EGFP. Data are shown as mean ± SEM of three independent assays. One-sample *t*-test was performed to analyze each average data above the predetermined threshold value of 80 (black dotted line), and *P* < 0.05 was considered statistically significant. ∗∗*P* < 0.01, ∗∗∗*P* < 0.001.

As shown in Figure 2, our assay detected the expected PIP-protein interactions with remarkable specificity: AKT-PH for PI(3,4,5)P_3_, PLCδ-PHx2 for PI(4,5)P_2_, and p40PHOX-PX for PI(3)P. A recent study (17) reported using SnxA as a specific reporter for PI(3,5)P_2._. We indeed observed strong binding of PI(3,5)P_2_ by SnxA, but a consistent, albeit modest, binding to PI(4,5)P_2_ was also detected.

### Detection threshold of lipid-SiMPull assay with Nanodiscs

**Fig. 3:**
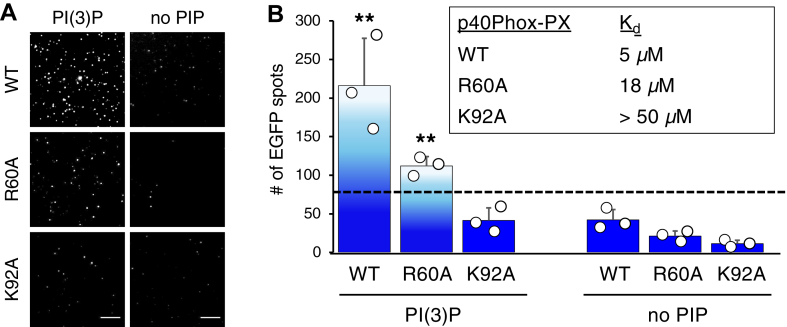
Detection threshold of Nanodisc-SiMPull assay. EGFP-p40PHOX-PX and mutants were transiently expressed in HEK293 cells, and cell lysates (5 nM EGFP-fusion) were subjected to SiMPull assay with 5% PI(3)P and negative control (no PIP) Nanodiscs. A: representative EGFP images are shown. Scale bars represent 10 μm. B: the numbers of adjusted EGFP spots are shown as mean ± SEM (n = 3 independent experiments). Reported *K*_d_s for PI(3)P (25) are shown in the inset. One-sample *t*-test was performed to analyze each average data above the predetermined threshold value of 80 (black dotted line), and *P* < 0.05 is considered statistically significant. ∗∗*P* < 0.01.

Previously, we had taken advantage of the known range of affinities of WT and mutant p40PHOX-PX for PI(3)P (25) to determine the detection limit of lipid-SiMPull assay using SUVs (8). Here, we used the same strategy to assess the assay with Nanodiscs. Representative TIRF microscopy images of the assay are shown in Figure 3A. WT and R60A, but not K92A, displayed above threshold and statistically significant binding to Nanodiscs containing 5% PI(3)P; none of these proteins bound Nanodiscs that did not contain PIP (Fig. 3B). Based on the reported affinities of these proteins for PI(3)P (Fig. 3B, inset), we conclude that the Nanodisc-SiMPull assay can detect PIP-protein interactions with affinity as low as *K*_d_ in the 10–20 μM range, similar to SiMPull assay using SUVs (8) and sufficient for detection of the vast majority of protein-PIP interactions known to be specific.

### Stoichiometry of PIP-protein interaction on Nanodiscs

**Fig. 4:**
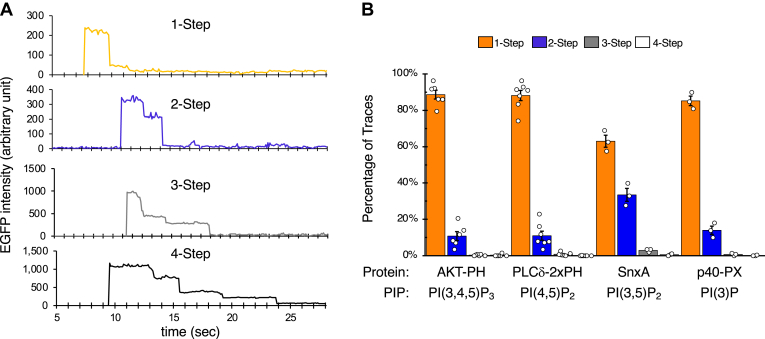
Stoichiometry of PIP-protein interactions on Nanodiscs. EGFP-fusion proteins were transiently expressed in HEK293 cells, and cell lysates (5 nM EGFP-fusion) were subjected to SiMPull assays using Nanodiscs containing 5% PI(3)P, 5% PI(4,5)P_2_, 5% PI(3,5)P_2_, or 10% PI(3,4,5)P_3_. A: For each protein-lipid binding pair, 30-40-s movies were captured, with initial excitation at 638 nm for visualization of DiD on Nanodiscs (not shown), followed by excitation at 488 nm for visualization of EGFP. Examples of single EGFP traces representing 1-, 2-, 3-, and 4-photobleaching steps are shown. B: Percentage of EGFP spots that displayed each of the four photobleaching steps is shown as mean ± SEM (n = 3–7 independent assays).

To determine the number of protein molecules bound on a single Nanodisc, we performed EGFP photobleaching analysis. Movies of 30–40 s in length were captured, with excitation at 638 nm for visualization of the Nanodiscs (DiD), followed by excitation at 488 nm for visualization of EGFP. Photobleaching steps of EGFP were analyzed for hundreds of Nanodiscs bound by EGFP for each protein-lipid pair (see examples in Figure 4A). Three of the four protein-lipid interacting pairs displayed photobleaching profiles similar to that of monomeric EGFP previously reported (7), with mostly 1-step bleaching (Fig. 4B). SnxA binding to PI(3,5)P_2_, however, displayed a marked degree of 2-step bleaching but not to the same extent of a tandem EGFP dimer (7). It has been reported that SnxA dimerizes via its coiled-coil domain (17). Therefore, the two-step bleaching we observed is likely from SnxA dimer rather than two SnxA monomers bound to each disc. Taken together, our observations suggest that each Nanodisc is bound by only one protein molecule or protein complex, conferring true single-molecule resolution.

### Characterization of transient interactions

**Fig. 5:**
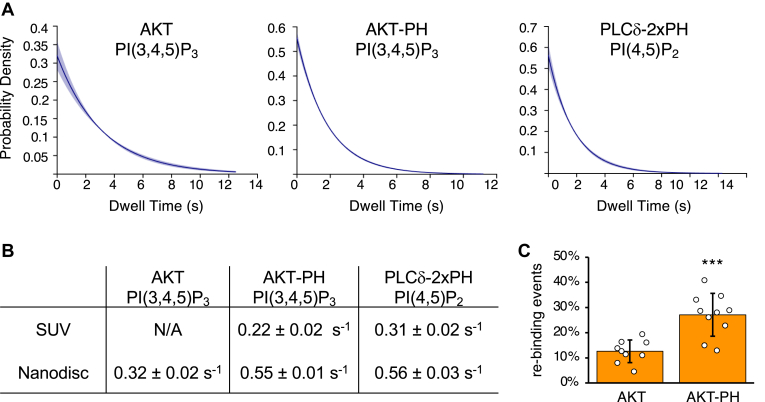
Characterization of transient interactions. EGFP-fusion proteins were transiently expressed in HEK293 cells, and cell lysates (5 nM EGFP-fusion) were subjected to Nanodisc-SiMPull assays. The Nanodiscs contained 5% PI(4,5)P_2_ or 10% PI(3,4,5)P_3_. A: Dwell time data were fit to exponential decay using a noncensored MLE model to yield dissociation rate constants in B. Shaded areas represent the 95% confidence intervals. B: Dissociation rate constants (*k*_off_) of the indicated protein-lipid pairs derived from SiMPull assays. SUV data are previously reported (7). C: Percentage of PI(3,4,5)P_3_ Nanodiscs displaying rebinding by AKT or AKT-PH is shown as mean ± SEM (n = 9–10 independent experiments). Unpaired *t*-test was performed to compare the data, and *P* < 0.05 is considered statistically significant. ∗∗∗*P* < 0.001.

Previously, we had reported characterization of dissociation rates (*k*_off_) for transient binding events in lipid-SiMPull assay using SUV (7). Here, we selected two lipid-protein pairs known to exhibit transient interactions—AKT-PH binding to PI(3,4,5)P_3_ and PLCδ-2xPH to PI(4,5)P_2_—and analyzed their dwell time in the Nanodisc-based SiMPull assay (Fig. 5A) to calculate *k*_off_. As shown in Figure 5B, the off rates were higher than those obtained previously with SUV-based SiMPull assay (7).

We had reported that AKT-PH displayed both stable and transient events of binding to PIP_3_-SUVs, whereas full-length AKT bound only stably (7). Under the microscope, transient binding of AKT-PH to SUVs was seen as rapid “blinking” of EGFP signals due to dissociation and rebinding of the protein. In the assay with Nanodiscs, however, we observed the blinking for both AKT-PH and full-length AKT. We set out to quantify the rebinding events and found that ∼13% of AKT and ∼30% of AKT-PH fluorescent spots displayed rebinding (Fig. 5C). In addition, the off rate of AKT in the transient binding events was markedly lower than that of AKT-PH (Fig. 5B). Taken together, protein-PIP interactions appear to be less stable with Nanodiscs than with SUVs. This is consistent with a stabilizing effect of PIP clustering on SUVs (26) and/or an underestimated dissociation rate due to multiple binding/rebinding events on a single SUV indistinguishable from long dwell times.

### Investigation of domains involved in stabilizing the AKT-PI(3,4,5)P_3_ interaction

**Fig. 6:**
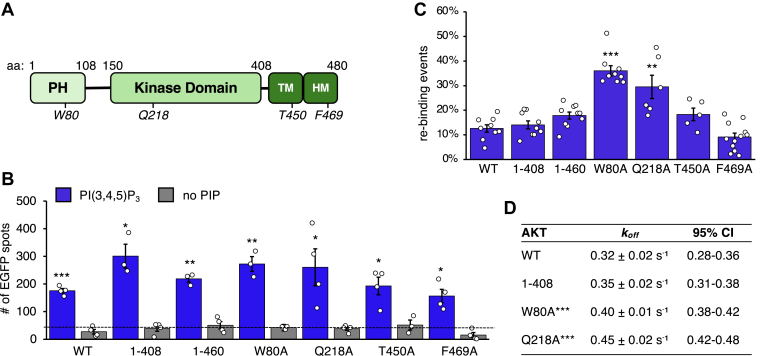
KD may contribute to stabilizing AKT interaction with PI(3,4,5)P_3_. A: Domain structure of AKT1 protein. Mutations characterized in this study are indicated. B: HEK293 cells transiently expressing EGFP-AKT and mutants were lysed, and lysates containing 5 nM EGFP-fusion proteins were subjected to Nanodisc-SiMPull assays. The Nanodiscs contained 10% PI(3,4,5)P_3_. The numbers of adjusted EGFP spots are shown as mean ± SEM (n = 3–4 independent experiments). One-sample *t*-test was performed to analyze each average data above the predetermined threshold value of 80 (black dotted line), and *P* < 0.05 was considered statistically significant. ∗*P* < 0.05, ∗∗*P* < 0.01, and ∗∗∗*P* < 0.001. C: Percentage of PI(3,4,5)P_3_ Nanodiscs displaying rebinding by AKT or mutants is shown as mean ± SEM (n = 5–10 independent experiments). Unpaired *t*-test was performed to compare the data, and *P* < 0.05 is considered statistically significant. ∗∗*P* < 0.01, ∗∗∗*P* < 0.001. D: Dissociation rate constants were determined for AKT and mutants as described in Fig. 5. Statistical analyses of comparison between mutants and WT are shown in supplemental Fig. S2. ∗∗∗*P* < 0.001. TM, turn motif.

The AKT signaling network is involved in the regulation of a broad spectrum of cellular processes from cell proliferation, differentiation, survival, and metabolism, and its dysregulation is connected to numerous human diseases (27). Intramolecular interactions involving the PH domain at the N terminus and the hydrophobic motif (HM) domain and a turn motif domain at the C terminus (Fig. 6A) keep the AKT kinase inactive before stimulation (28, 29). This autoinhibition can be relieved by PH domain binding to PIP_3_ (30) and recruitment of AKT to the plasma membrane, where the activation loop (T308) and HM (S473) are phosphorylated by PDK1 and mTORC2, respectively, leading to activation of the kinase (27, 28, 29). mTORC2 also phosphorylates T450 in the turn motif to stabilize the AKT protein (27). How PH domain interaction with PIP_3_ may be modulated by other domains of AKT has not been extensively studied.

We found the difference between AKT and AKT-PH in the stability of their binding to PIP_3_ intriguing and set out to probe the possible involvement of domains outside PH. To examine potential contribution of the C terminus to the stability of AKT-PIP_3_ interaction, we expressed AKT mutants with various lengths of C-terminal deletion and subjected them to Nanodisc-SiMPull assays. All mutants displayed above-threshold binding to PIP_3_-containing Nanodiscs (Fig. 6B), and rebinding events on the discs occurred for these mutants to an extent comparable to that of the WT-AKT (Fig. 6C). We also found *k*_off_ of the interaction for aa1-408-AKT to be 0.35 s^−1^, comparable to that of WT (0.32 s^−1^) and markedly lower than that of PH domain alone (0.55 s^−1^) (Figs. 5B, 6D and supplemental Fig. S2). Therefore, it does not appear that the C terminus of AKT is involved in stabilizing PIP_3_ binding of the PH domain.

With the C terminus ruled out, the kinase domain (KD) became the primary candidate for contributing to the stability of PH-PIP_3_ interaction. We decided to evaluate W80 in the PH domain, because *a*) this residue did not appear to be directly involved in the binding of PIP_3_ as deduced by the crystal structure of AKT-PH and IP_4_ complex (31) and *b*) W80 was suggested to mediate PH-kinase interaction by inserting into a deep cleft in the KD (32). As expected, the W80A mutation did not affect steady-state binding to PIP_3_-containing Nanodiscs by full-length AKT (Fig. 6B). However, W80A-AKT exhibited increased rebinding (Fig. 6C) and *k*_off_ (Fig. 6D), both reflecting decreased dwell time in PIP_3_ binding. These observations suggest that interaction with the KD may stabilize PH domain’s binding to PIP_3_. A residue in the KD, Q218, was previously found to interact with phosphorylated S473 in the C-terminal HM domain, which is necessary for AKT activation (33, 34). Interestingly, Q218A-AKT also displayed increased rebinding to PIP_3_ Nanodiscs (Fig. 6C) and a drastically higher *k*_off_ compared with WT-AKT (Fig. 6D and supplemental Fig. S2). We do not have a definitive explanation for this behavior of Q218A. However, given that the involvement of S473 has been ruled out by our observations with the AKT construct containing only amino acids 1–408, we are inclined to speculate that either Q218 is directly involved in an interaction between KD and PH, or Q218A induces a conformational change that indirectly affects KD-PH interaction. Two other residues were also examined in our assays: F659, which is suggested to interact with W80 (33), and T450, phosphorylation of which stabilizes the AKT protein (27). Neither F659A nor T450A affected the occurrence of rebinding by AKT to PIP_3_ (Fig. 6B–D), consistent with the observation that deletion of the entire C terminus did not affect the stability of AKT-PIP_3_ interaction.

## Discussion

We have demonstrated that replacing SUVs with Nanodiscs retains the detection threshold and specificity of the lipid-SiMPull assay in studying PIP-protein interactions. Most importantly, we find that each Nanodisc provides one binding site for a PIP-interacting protein, conferring single-molecule resolution that is not possible with SUVs. Our 10-nm Nanodiscs had a lipid surface area of ∼40 nm^2^ (as opposed to 2,000–8,000 nm^2^ for 50–100 nm SUVs), which likely precluded simultaneous binding of multiple copies of an average-sized protein. In addition, the probability of more than one protein occupying each Nanodisc is low at 5 nM of the target protein in our assay. The single-molecule detection capability of the Nanodisc-SiMPull assay is a major advantage over the use of SUV in the assay.

Our new assay enables determination of dissociation rate constants of PIP-protein interactions at the single-molecular level, which are found to be higher than the rates obtained using SUVs. This difference could be due to the stabilizing effect of PIP clustering on SUV, a well-known phenomenon (26), which may be absent on a Nanodisc that contains only three to eight PIPs at 5–10%. Another likely possibility is that binding/rebinding to the same SUV by multiple copies of the protein would occur due to the high number of PIPs on the vesicle, which would not be distinguishable from long dwell times of a single protein by TIRF microscopy, resulting in an underestimated dissociation rate in the SUV-SiMPull assay. The small but not insignificant fraction of AKT molecules that exhibit transient binding to PI(3,4,5)P_3_ was previously missed and now captured by the Nanodisc-SiMPull assay, highlighting the unique advantage of this new method.

Utilizing the new assay we have searched for a mechanism underlying the transient nature of the AKT PH domain interacting with PI(3,4,5)P_3_ in contrast to the more stable binding by the full-length AKT protein. Our results suggest that a previously unidentified intramolecular interaction between the KD and the PH domain of AKT most likely contributes to stabilizing the lipid-protein interaction. Future structural and computational analyses are warranted to further define the relationship between the two domains in the membrane.

The simple set-up of the SiMPull assay offers a method of detecting protein-lipid interactions that can be adopted by many laboratories with relative ease. In addition to the single-molecule resolution, advantages of Nanodiscs include their stability, precisely defined size, and their flatness that makes them a better mimic for cell membranes (especially plasma membrane). The Nanodisc-SiMPull assay will enable accurate assessments of lipid-associated protein complexes in their composition and stoichiometry. Another unique feature of the Nanodisc is its capacity to embed proteins of interest, such as transmembrane receptors and peripheral membrane-associated proteins (14). Therefore, our new assay can be employed to investigate the formation of signaling complexes involving cooperative lipid-protein and protein-protein interactions.

## Data availability

Original data are available from the lead contact upon request. The IDL and MATLAB scripts used in this study are available at GitHub and Zenodo. smFRET package used for SiMPull image acquisition: https://github.com/Ha-SingleMoleculeLab; IDL scripts used to process raw image files: https://doi.org/10.5281/zenodo.4925617; scripts for maximum likelihood estimation and LRT analyses: https://doi.org/10.5281/zenodo.15313208. Any additional information required to reanalyze the data reported in this article is available from the lead contact upon request.

## Supplemental data

This article contains supplemental data.

## Conflict of interest

The authors declare that they have no conflicts of interest with the contents of this article.
