# Supplementary material for: Nanodisc single-molecule pulldown to study lipid-protein interactions
Source: J Lipid Res. 2025 Jun 20;66(7):100846. doi: 10.1016/j.jlr.2025.100846 (PMC12284033; doi:10.1016/j.jlr.2025.100846)
Supplement: Supplemental Figures [file mmc1.pdf]

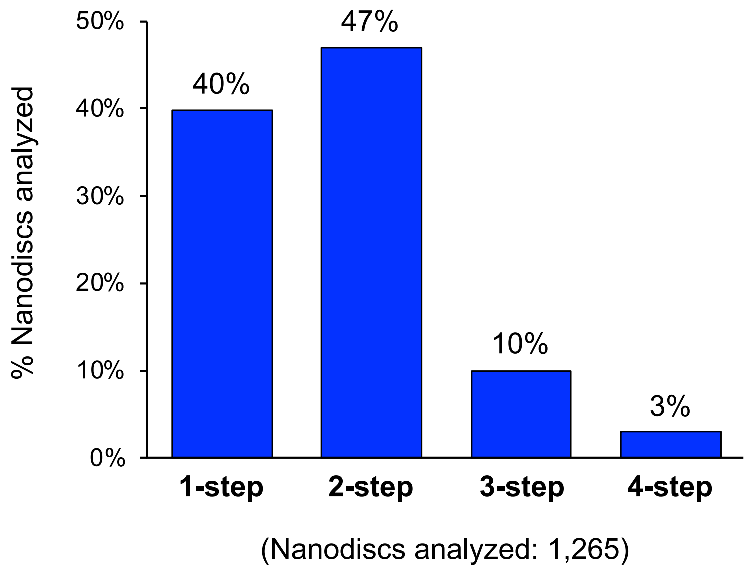

Fig. S1. Photobleaching step analysis to determine copy number of DiD incorporated into Nanodiscs. DiD was incorporated into PIP_3-containing biotinylated Nanodiscs as described in Materials and Methods. The discs were then pulled down via biotin onto slides for TIRF microscopy to analyze photobleaching steps. The result is consistent with 1 to 4 copies of DiD per Nanodisc, with the majority of discs containing 2 copies of DiD. A combination of 1-step and 2-step photobleaching events is expected when two DiD molecules are on the same disc.

A

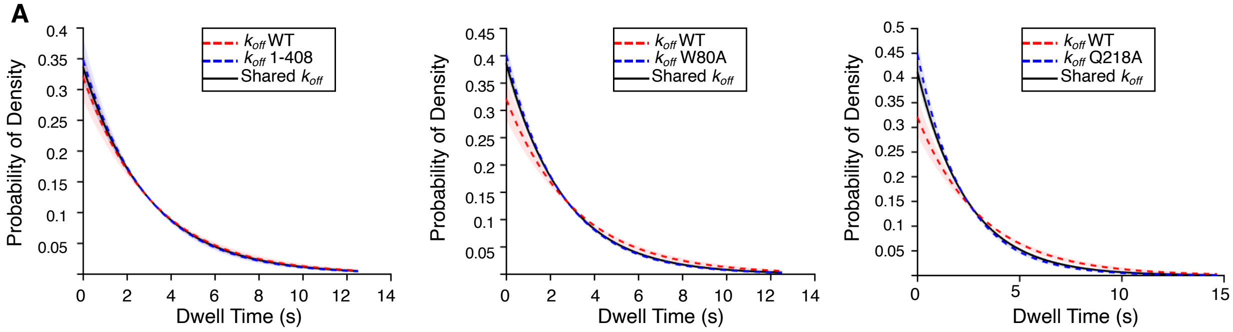

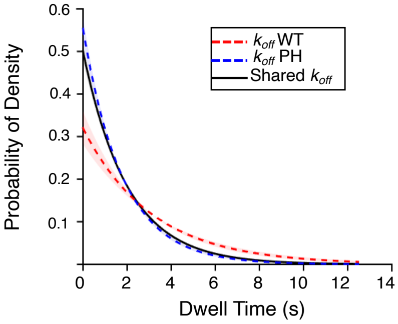

B

vs WT	Shared k_{off}	Chi-square (χ^2)	p Value	Comparison
AKT-PH	0.501 ± 0.012 s^{-1}	77.03	1.8×10^{-18}	Significant
AKT 1-408	0.336 ± 0.013 s^{-1}	1.07	0.3	Not Significant
W80A	0.385 ± 0.010 s^{-1}	11.81	5.9×10^{-4}	Significant
Q218A	0.411 ± 0.012 s^{-1}	24.58	7.13×10^{-7}	Significant

Fig. S2. Comparison of k_{off} using LRT analysis. Likelihood ratio test (LRT) was used to statistically compare k_{off} between WT-AKT and each mutant. Chi-square distribution with 1 degree of freedom was used to determine the significance ($\chi^2 = 3.84$ for $p = 0.05$). **(A)** Dwell time data for WT AKT (dashed red) and mutant (dashed blue) were fitted to exponential decay independently. The combined data (solid black) was fitted under the assumption that the two datasets had the same dissociation rate. Shaded areas represent 95% confidence intervals for each fit. **(B)** Summary of LRT results from data in A. Significant difference is indicated by $p < 0.05$.
